# Obesity Awareness and Sensitization in Schools for Improving Weight Control and Reducing Obesity Stigma in Students: A Pilot Study

**DOI:** 10.7759/cureus.71910

**Published:** 2024-10-20

**Authors:** Indu Saxena, Manoj Kumar, Alka Shukla, Rajshri Aishwarya, Ashish Arvind, Aniruddha Sen, Babu L Kannoujia

**Affiliations:** 1 Biochemistry, All India Institute of Medical Sciences Gorakhpur, Gorakhpur, IND; 2 Physiology, Maharshi Vashishtha Autonomous State Medical College, Basti, Basti, IND; 3 Pediatrics, Maharshi Vashishtha Autonomous State Medical College, Basti, Basti, IND; 4 Medical Education, Uttar Pradesh University of Medical Sciences Saifai, Saifai, IND; 5 Physiology, All India Institute of Medical Sciences Gorakhpur, Gorakhpur, IND; 6 General Medicine, Maharshi Vashishtha Autonomous State Medical College, Basti, Basti, IND

**Keywords:** medical education, obesity bias, obesity destigmatization, pediatric obesity, school students

## Abstract

Introduction: The prevalence of obesity is increasing rapidly in the world, including India. Since obesity is a risk factor for many diseases, the prevalence of obesity-related diseases is also increasing, resulting in decreased productivity and increased disease-related expenses, causing economic loss at the individual, family, and national levels. Government funds that could be utilized for national growth are channeled into the treatment of non-communicable diseases (NCDs).

The prevalence of childhood overweight and obesity has also increased in recent years. Overweight or obese children often grow up to become overweight or obese adults. While the treatment of childhood obesity is gaining importance, the general population is still unaware of the seriousness of this condition. The government of India has been promoting a healthy diet and yoga to prevent or delay the onset of NCDs.

Methods: In this pilot study, we reached out directly to the affected persons: the children. We focused on students of class eight who have studied nutrition in school and are young enough to develop a lifelong healthy lifestyle to regulate body weight. Our obesity awareness and sensitization program (OASP) included a questionnaire assessing students’ awareness about healthy body weight. This was followed by three lectures on the causes and consequences of obesity and difficulty in achieving weight loss. Since overweight or obese children may experience weight-related bias, teasing, and bullying in schools, the OASP also addressed obesity bias. Pre- and post-tests were conducted to assess explicit obesity bias before and after the OASP. Data obtained from 389 students was used. The pre- and post-test results were compared using Student's t-test.

Results: About 27.8% of students were overweight or obese. Most participants of this study were not aware of healthy body weight, diseases associated with obesity, and the difficulty of losing weight. Many students expressed explicit bias against obesity. The OASP increased the students’ knowledge about ideal weight, healthy diet, and the importance of lifestyle changes. Explicit obesity bias showed a significant decrease after the program.

Conclusion: This study showed that most students of class eight who participated in this study were not aware of the importance of healthy body weight, obesity-related diseases, healthy lifestyles for long-term regulation of healthy body weight, and the negative consequences of obesity bias. The OASP addressed all these issues and was found successful.

## Introduction

Obesity is a chronic, relapsing, usually multifactorial condition resulting from excess or abnormal deposition of body fat [1]. According to the World Health Organization (WHO), adults with body mass index (BMI) values higher than 25 kg/m2 are considered overweight, while persons with BMI greater than 30 kg/m2 are considered obese. However, these values are different for different races. In general, fat content that is higher than 20% of normal according to height is often associated with an increased risk of obesity-associated diseases. In the case of children aged 5-19 years, a BMI age greater than 1 standard deviation above the WHO Growth Reference median is considered overweight, and a BMI age greater than 2 standard deviations above the WHO Growth Reference median is considered obese [2].

More than two billion adults (44% of the global adult population) were reported to be overweight or obese in 2016, of whom more than 70% were from low- or middle-income countries [3]. The prevalence of obesity in India (as determined by the BMI) was 13.85%, while the prevalence of central obesity was 57.71% [4]. Central obesity, which is more common in Asian Indians [5], is associated with insulin resistance that can lead to type 2 diabetes mellitus, cardiovascular diseases (CVDs), dyslipidemia, non-alcoholic steatohepatitis, polycystic ovarian syndrome, obstructive sleep apnea, certain cancers, etc. This relationship between abdominal obesity and metabolic diseases was recognized more than three decades ago, and the use of waist circumference as an indicator of abdominal obesity was suggested in 1997 [6]. Obesity lowers the productivity of the individual, and obesity-related diseases pose an economic burden on the family as well as the nation [7].

The prevalence of overweight and obesity in India is increasing at a rate faster than in the rest of the world, due to the growth of the socioeconomic sector, rapid urbanization, and resultant lifestyle changes. According to a meta-analysis published in 2023, more than 12% of children were overweight, while more than 8% were obese [8]. Childhood overweight and obesity need to be tackled early, before the onset of obesity-related complications like hypertension, early-onset type 2 diabetes, pain in weight-bearing joints, PCOS, etc. [9].

Current practices regarding chronic diseases (like CVDs and type 2 diabetes) usually involve a focus on diagnosis and treatment of the disease, with little or no emphasis on prevention. The situation can be controlled better by contacting the target population directly and spreading awareness about the condition, its identification, and prevention. The government of India has focused on the prevention of lifestyle diseases by promoting healthy lifestyles and yoga. Children and adolescents need encouragement and motivation to adopt a healthier lifestyle and to have healthier citizens in the future so that the expenditure on non-communicable diseases may be channeled into projects leading to national development.

Besides being at higher risk for obesity-related diseases, obese persons often face discrimination in society. While discrimination on the basis of gender, race, color, and religion is considered an inexcusable offense, discrimination against obese persons is often overlooked. Consequently, obese persons often face stressful situations or experience bias in social encounters, including job opportunities. Obese children may also experience bias. This bias may be explicit (conscious preference or aversion toward a particular group of persons, usually fewer in number) or implicit (the person may be unaware of the bias). Explicit bias is often expressed in the form of negative remarks, teasing, bullying, etc. Implicit bias results in automatic reactions. Many children who are overweight or obese are targets of weight-related teasing and bias [10-12]. Such children may experience stress that may lead to increased appetite with further aggravation of the condition. Obesity bias can be overcome by providing information about obesity, its causes, and the consequences of obesity bias in society. It is important to remove obesity-related stigma from the minds of children and also to help children in handling such stigma.

We have been unable to find publications reporting obesity awareness, sensitization, or obesity bias assessment in schoolchildren. In India, students of the eighth standard are familiar with the concepts of nutrition, the calorie content of food, healthy food habits, etc., and we, therefore, addressed these students in our study. The objectives of this study were to increase awareness about healthy weight and lifestyle, measure explicit obesity stigma by examining anti-fat attitudes and beliefs about obese persons, and examine change in anti-fat attitudes and beliefs about obese persons after conducting a program on obesity awareness and destigmatization.

## Materials and methods

This pilot study was conducted after obtaining permission from the Institute Human Ethics Committee of All India Institute of Medical Sciences Gorakhpur (IHEC ref number: IHEC/AIIMS-GKP/BMR/32/2020, dated March 21, 2022). Permission was also obtained from the principal/director of the school/institute where the study was conducted. Students and their parents were informed in advance regarding this activity through individual notes sent to the parents under the standard school/coaching institute protocol. Any students who did not wish to participate could absent themselves. The inclusion criteria for the study required students to be enrolled in class eight of two schools and one coaching center. The exclusion criteria specified that any student who did not wish to participate would not be included. Table 1 provides a summary of the details of the participating students.

**Table 1:** Details of student participants All students were enrolled in Class VIII of their respective school/institute. F: female, M: male, T: total (female + male)

School		I			II			III			All schools		
		F	M	T	F	M	T	F	M	T	F	M	T
Students enrolled		82	93	175	54	62	116	39	93	132	175	248	423
Absent/incomplete forms		6	2	8	6	2	8	8	10	18	20	14	34
Complete forms		76	91	167	48	60	108	31	83	114	155	234	389
Age	Mean	12.66	12.68	-	12.63	12.83	-	13.90	13.94	-	12.9	13.17	-
	SD	0.58	0.64	-	0.63	0.71	-	0.69	0.70	-	0.80	0.89	-
	p-value	-	0.81	-	-	0.12	-	-	0.81	-	-	0.002	-

A total of 423 students were enrolled in the eighth standard of the three schools/institutes. Thirty-four students were either absent or submitted incomplete questionnaires or modules for measurement of explicit bias. Thus, questionnaires and modules obtained from 389 students (155 female) were used.

The study was conducted separately in each school or institute and involved three visits to each school or institute. On the first day of the visit, the students were given a 20-point questionnaire and pre-test modules for measurement of explicit obesity bias. After collecting the responses from the students, the first session of the obesity awareness and sensitization program (OASP) was held. The next two sessions of OASP were held on consecutive days. The post-test modules for explicit obesity bias were distributed at the end of the OASP on the third day, and responses were collected after 15 minutes.

Record of height and weight

Students recorded their height and weight under the guidance of their teachers on standardized and calibrated equipment provided by us before initiating the study program. Body weight was recorded after removing shoes, belts, and heavy clothing on digital weighing scales with a least count of 100 grams. Height was recorded (without shoes) in centimeters using a stadiometer.

Questionnaire and pre-test measurement of obesity bias

A questionnaire with 20 multiple-choice questions was filled out by the students before the OASP. The first 16 questions required marking the single best response. The first 10 questions were related to awareness about overweight and obesity, and the next six questions were related to weight reduction and associated problems. Four questions that were related to the causes and consequences of obesity could have more than one response.

Explicit bias against obesity was measured using two simple modules: the Anti-fat Attitude Scale (AFAS) and the Beliefs About Obese Persons (BAOP) Scale [13,14]. The AFAS has five statements that are marked on the Likert scale by the participant, ranging from strongly disagree (score 1) to strongly agree (score 5). The highest possible score is 25, while the lowest possible score is 5. Higher scores indicate higher anti-fat attitudes. The BAOP Scale has eight statements that are marked on the Likert scale, ranging from strongly disagree to strongly agree. The possible total ranges from 0 to 48. Higher scores indicate a stronger belief that obesity is not under a person’s control. Both of these modules were first tested on a sample of 25 students of the same standard and age group and were found to be comprehensible and easy to answer.

Although the schools were either English-medium or had advanced English as a subject, some students were not fluent in the language. For such students, the questionnaires and modules were translated and printed in Hindi. These were tested on both faculty and school students to ensure equivalence.

OASP

This was conducted over three consecutive days and included three lectures (each of one-hour duration) on the causes and consequences of obesity, difficulties in losing weight, and obesity stigma and its consequences. The language of lectures was both English and Hindi. Students were informed about reading nutritional labels on food items and interpreting the sugar and fat content of foods. Metabolic consequences of high fructose and trans fats were discussed. Questions were asked during and at the end of the program to test the understanding of the students.

Post-test measurement of obesity bias

Changes in explicit bias on attending the OASP were again measured on the AFAS and BAOP Scale on the last day of the session. Pre- and post-test results were compared separately for each module.

Data analysis

The results of the questionnaire were calculated in terms of percentages. Results of each module for explicit obesity bias were analyzed separately by Student’s t-test to infer the effect of OASP. A paired t-test was used for the comparison of pre- and post-test scores in the same category (female or male), while an unpaired t-test was used to compare pre-test scores and post-test scores of female and male students.

## Results

Table 2 summarizes the responses obtained for the first 10 questions related to awareness about overweight and obesity. The student had to tick the single best response for each question. None of the students were aware of BMI. Four female (out of 155) and five male students (out of 234) believed themselves to be underweight. The formula for the calculation of BMI and the WHO Growth Reference Charts for Children and Adolescents aged 5-19 years were displayed on the screen for the students to assess their own weight. After calculation of the actual BMI from the formula provided and the BMI charts for female and male adolescents, only one male student was found to be actually underweight. Forty-nine female and 34 male students (a total of 19.8%) were overweight, while nine female and 23 male students (a total of 8%) were obese. The table shows the percentage of students in each category.

**Table 2:** Summary of responses on questionnaire related to awareness about overweight and obesity. Single best response was marked by the student participant N: total number of students in the category (female, male, and both female + male), n: number of persons giving a specific response

SN	Question	Response	Female students (N=155)		Male students (N=234)		All students (N=389)	
			n	%	n	%	n	%
1.	Are you aware of body mass index (BMI)?	Yes	0	0	0	0	0	0
		Not sure	9	5.81	27	11.54	36	9.25
		No	146	94.19	207	88.46	353	90.74
2.	In your opinion, what is your current weight status?	Underweight	4	2.58	5	2.14	9	2.31
		Normal	115	74.19	204	87.18	319	82.00
		Overweight	29	18.71	19	8.12	48	12.34
		Obese	7	4.52	6	2.56	13	3.34
3.	On the basis of the BMI formula provided and the chart shown on the screen, what is your actual weight status?	Underweight	0	0	1	0.43	1	0.26
		Normal	103	66.45	176	75.21	279	71.72
		Overweight	49	31.61	34	14.53	77	19.79
		Obese	9	5.81	23	9.83	32	8.23
4.	Is there any family member who is overweight or obese?	Yes	16	10.32	28	11.97	44	11.31
		No	139	89.68	206	88.03	345	88.69
5.	Do you have any relatives who are overweight or obese?	Yes	94	60.64	133	56.84	227	58.35
		No	61	39.35	101	43.16	162	41.64
6.	Do you have any friends who are overweight or obese?	Yes	30	19.35	72	30.76	102	26.22
		No	125	80.64	162	69.23	287	73.78
7.	Do you have any acquaintances who are overweight or obese?	Yes	155	100	234	100	389	100
		No	0	0	0	0	0	0
8.	Have you ever used weight-related nicknames while addressing others?	Yes	134	86.45	223	95.3	357	91.77
		No	21	13.55	11	4.7	32	8.23
9.	Do you or your friends comment on or criticize the weight of persons with obesity?	Yes	116	74.84	179	76.5	295	75.84
		No	39	25.16	55	23.5	94	24.16
10.	Do your family members comment on or criticize the weight of persons with obesity?	Yes	127	81.94	204	87.18	331	85.09
		No	28	18.06	30	12.82	58	14.91

Although only 44 students out of 389 reported having an immediate family member who is overweight or obese, more than 58% of students reported having relatives who are overweight or obese. All students reported having an acquaintance with overweight or obesity, but only 26% of students had friends with this condition. Most of the students reported that they, their friends, or family members had used weight-related nicknames to describe persons with obesity, had made negative comments related to such persons, or had criticized persons with obesity for having this condition.

Table 3 shows the responses obtained for the next six questions related to weight reduction. We wanted to assess if the students were aware of the difficulties in losing weight. In this section also, the students were required to mark the single best option. Many students had either attempted to lose weight themselves or had a relative or friend who had tried to lose weight. Only 17.3% reported success in weight loss endeavors (of either themselves or their relative/friend).

**Table 3:** Summary of responses related to questionnaire related to weight reduction and associated problems. Single best response was marked by the student participant N: total number of students in the category (female, male, and both female + male), n: number of persons giving a specific response

SN	Question	Response	Female (N=155)		Male (N=234)		Total (N=389)	
			n	%	n	%	n	%
1.	Have you/your family members attempted to lose weight?	Yes	57	36.77	70	29.91	127	32.65
		No	98	63.22	164	70.08	262	67.35
2.	To your knowledge, has any friend/relative attempted to lose weight?	Yes	99	63.87	109	46.58	208	53.47
		No	56	36.13	125	53.42	181	46.53
3.	If the answer to either of the above two questions is yes, were you/your family member/relative/friend successful in reducing weight?	Yes	7	12.28	15	21.43	22	17.32
		No	50	87.72	55	78.57	105	82.68
4.	Which, in your opinion, is the easiest method to lose weight?	Reducing the size of meal	57	36.77	61	26.07	118	30.33
		Skipping meals	39	25.16	60	25.64	99	25.45
		Omitting high-calorie food	45	29.03	49	20.94	94	24.16
		Increasing physical activity	14	9.03	64	27.35	78	20.05
5.	In your opinion, when should a person become aware of their higher-than-normal weight status and try to reduce it?	As early as possible (parents should intervene in case of children)	4	2.58	9	3.85	13	3.34
		After puberty (age 10-12 years)	15	9.68	52	22.22	67	17.22
		Early adulthood (age 18-25 years)	55	35.48	100	42.73	155	39.84
		On onset of obesity-related health problems	81	52.26	73	31.20	154	39.59
6.	Constantly reminding a person with obesity to reduce food and increase physical activity can lead to:	Weight reduction	92	59.35	132	56.41	224	57.58
		Depression, with increased appetite	58	37.42	90	38.46	148	38.05
		No effect	5	3.22	12	5.13	17	4.37

Reducing the size or number of meals was considered an easy method of losing weight. Most students believed that weight loss interventions should be adopted after attaining adulthood or after the onset of obesity-related diseases. A large number of students (57.58%) believed that constantly reminding a person with obesity about their condition would help in weight reduction.

Responses to the last four questions of the questionnaire are summarized in Table 4. These questions were related to the causes and consequences of obesity. Each of these questions could have more than one response. The students were asked to mark all the options they considered correct. Most students were aware that hypertension and diabetes mellitus are related to obesity.

**Table 4:** Summary of responses related to questionnaire related to causes and consequences of obesity. Students could mark more than one option N: total number of students in the category (female, male, and both female + male), n: number of persons giving a specific response

SN	Question	Response	F (N=155)		M (N=234)		T (N= 389)	
			n	%	n	%	n	%
1.	Which of these conditions is usually the cause of overweight or obesity?	Large appetite	152	98.06	188	80.34	340	87.40
		Certain genetic conditions	11	7.10	22	9.40	33	8.48
		Certain hormone imbalances	37	23.87	34	14.53	71	18.25
		Lack of physical activity	52	33.55	85	36.32	137	35.22
		Other reasons	3	1.93	5	2.14	8	2.06
2.	Being overweight is not good for a person because:	Affects looks, and decreases self-confidence	151	97.41	140	59.83	291	74.81
		Increases risk of certain other diseases	67	43.22	121	51.71	188	48.33
		Person eats more than a fair share	3	1.93	2	0.85	5	1.28
		Decreases activity, efficiency, intelligence	34	21.93	71	30.34	105	26.99
3.	Underweight is not good for a person because:	Decreases capacity to do physical work	73	47.1	139	59.40	212	54.50
		Risk factors for other diseases	97	62.58	124	52.99	221	56.81
		Shows apathy toward food and life	4	2.58	1	0.43	5	1.28
4.	Which of the following diseases can be related to overweight or obesity:	Hypertension	103	66.45	172	73.50	275	70.69
		Diabetes mellitus	150	96.77	165	70.51	315	80.98
		Pain in joints	57	36.77	52	22.22	109	28.02
		Problems associated with the reproductive system	0	0	4	1.71	4	1.03
		Breathing problems	0	0	26	11.11	26	6.68

The three lectures on obesity awareness and sensitization were interspersed with questions for the audience. Verbal responses from the students showed their attentiveness and understanding. Faculty and non-teaching staff also participated in these verbal queries. The audience was encouraged to ask questions during the lectures. All students benefitted from this program and gave correct responses to the questions.

Table 5 shows the results of pre- and post-tests of the two modules on explicit obesity bias. The data were compared by a paired and unpaired Student’s t-test. Pre- or post-test scores of the same category (female or male) were compared by paired t-test, while pre-test scores of female and male students and post-test scores of female and male students were compared by unpaired t-test. The mean AFAS score was significantly lower in the post-test compared to the pre-test. Higher scores indicate higher anti-fat attitudes. The BAOP Scale score showed a significant increase in the post-test conducted after the OASP. Higher BAOP Scale scores indicate a stronger belief that obesity is not under the affected person’s control.

**Table 5:** Summary of the AFAS and BAOP results obtained before and after conduction of the obesity sensitization program AFAS: Anti-fat Attitude Scale, with five statements that are marked on the Likert scale. The scoring method is provided with the AFAS. The lowest score possible is 05 and the highest score possible is 25. Higher scores indicate higher anti-fat attitudes. BAOP Scale: Beliefs About Obese Persons Scale, with eight statements that are marked on the Likert scale. The scoring method is provided with the BAOP Scale. The lowest score possible is 00 and the highest score possible is 48. Higher scores indicate a stronger belief that obesity is not under the control of the person with obesity. * Paired t-test for comparison within the same group, $ Unpaired t-test for comparison between female and male students

		AFAS scores			BAOP Scale scores		
		Pre-test	Post-test	p-value	Pre-test	Post-test	p-value
Female (N=155)	Mean	15.813	10.922	<0.0001*	19.716	25.000	<0.0001*
	SD	2.821	2.694	-	2.154	3.131	-
Male (N=234)	Mean	18.718	15.718	<0.0001*	19.714	25.919	<0.0001*
	SD	2.253	3.260	-	3.113	3.871	-
	p-value (F-M)	<0.0001$	<0.0001$	-	0.993$	0.004$	-

## Discussion

The mean age of female students was 12.9 years, significantly lower than the mean age of the male students (13.17 years) (Table 1). Students enrolled in the coaching institute were slightly older than the students enrolled in regular schools.

Table 2 shows that while nine students (2.3%) considered themselves underweight, the actual number was only one (0.26%). Eighty-two percent of students (74.2% female, 87.2% male) had normal body weight. In this sample, the prevalence of overweight or obesity was 27.8%: 31.6% of females were overweight and 5.8% were obese, while 14.5% of males were overweight and 9.83% were obese.

Although all students (100%) had acquaintances who were overweight or obese, only 26.2% reported having a friend with this condition. This could be due to the relatively smaller number of overweight or obese children in this age group, or it could be due to obesity bias.

Most of the students (86% female and 95% male) reported using weight-related nicknames; 75.8% of students reported that they or their friends commented on the weight or criticized the weight of persons with obesity, showing the presence of obesity bias in schoolchildren. Eighty-five percent of students reported that their family members commented on their weight or criticized the weight of persons with obesity. This suggests that explicit obesity bias did not decrease with age and could be a learned behavior in the students participating in this study, as their family members also showed this behavior. Weight-related comments/nicknames/teasing were not considered wrong or offensive in these families or by the participating students. It also indicates the belief that a person’s weight was his/her choice and could easily be reduced.

Table 3 summarizes the responses of students to questions related to weight loss. Thirty-two percent of students or their family members had ever attempted to reduce their weight, while 53% had relatives or friends who had attempted to lose weight. Of these, only 17% of students reported that weight loss had occurred in the person concerned. These figures indicated that weight loss was not easy to attain.

Almost one-third of students (30.3%) believed that reducing the size of meals was the easiest method to lose weight. Twenty-five percent considered skipping meals as the easiest method, 24% had faith in omitting high-calorie food, and 20% considered increased physical activity as the easiest option. These results show that students were not aware of the difficulties in attaining weight loss. Almost 80% of students (more than 39% in each case) believed that weight reduction attempts should begin in early adulthood or on the onset of weight-related health problems. Only 3.3% believed that higher-than-normal weight in children required intervention. This attitude may result in the postponement of weight loss interventions at an early age, resulting in higher obesity rates. Weight loss interventions in children, under medical supervision, are required to control obesity.

More than half the students (57.58%) believed that constantly reminding a person with obesity to reduce diet and increase physical activity would lead to weight loss, while 38% felt that this could lead to depression with resultant weight gain. This suggests that weight-related teasing or remarks may be wrongly considered an attempt to make someone lose weight.

Most of the students (87.4% total) considered that being overweight or obese was caused by a large appetite (Table 4). Only 8.4% of students were aware of the genetic causes of obesity. This is because monogenic and syndromic obesity are not common. A few students (18.25% total, 23.9% female) were aware of the hormonal causes of overweight or obesity, especially hypothyroidism. Lack of physical activity was considered a common cause by 35% of students. Information regarding the causes and consequences of obesity was discussed in detail in the OASP [15].

Seventy-four percent of students felt that being overweight or obese affected the looks of a person and decreased their self-confidence. Weight-related teasing or bullying would further decrease the confidence of a person who is overweight or obese and should be discouraged. Forty-eight percent were aware that this condition increased the risk of other diseases. Alarmingly, 27% of students felt that being obese decreased the activity, efficiency, and intelligence of the person. However, according to the school teachers, most of the overweight or obese students were scoring high marks in various subjects.

Many students (54.5%) believed that being underweight reduced their capacity to do physical work, and 56.8% considered it a risk factor for other diseases. Only 1.2% believed that being underweight showed apathy toward food and life. While most students were aware that overweight and obesity are related to hypertension (70.7%) and diabetes mellitus (81%), few (28%) were aware that excess weight is a risk factor for joint pain. Only 1% of students (0% female) were aware that obesity is a risk factor for problems associated with the reproductive system (e.g., polycystic ovarian syndrome, infertility, etc.), and only 6.7% of students (0% females) were aware that obesity could cause respiratory problems.

The students' responses showed a clear majority or distinct grouping for most questions. For example, most students were unaware of BMI, had witnessed or participated in stigmatization, and believed that appetite was the primary cause of obesity. The responses did not follow a normal distribution and were heavily skewed in one direction, indicating significant polarization of opinion or awareness level due to unawareness and/or bias influenced by social norms.

The OASP included information on obesity-related diseases, associated productivity and economic loss, and the metabolic basis of why weight loss is difficult to achieve (the yo-yo effect). The lack of information regarding overweight and obesity, their causes, and consequences apparent from the students’ responses in the questionnaire showed the importance of conducting such programs in schools. Many of the teachers and non-teaching staff of the school also expressed ignorance related to the causes of obesity and the associated risk factors and were happy to attend this program.

A special volunteer movement was organized in Russia in 2013 for the prevention and timely detection of CVD [16]. The purpose of this movement was to involve students in volunteer activities to provide medical information and assistance to the general population. One goal of this movement was to popularize a healthy lifestyle and educate the public about the prevention of CVD. The movement gained momentum and popularity due to its performance, and on 21 March 2020, the all-Russian Mutual Aid Action (WeAreTogether) was launched. Similar programs can be beneficial in India, as these are cheap and easy methods of spreading information to a vast number of persons. Intensive behavioral interventions like supervised physical activity sessions, information about healthy diet, reading and interpretation of food labels, etc. can promote weight loss in children and teens [17]. The Stanford Youth Diabetes Coaches Program conducted for adolescents was found to be effective in underserved settings [18]. A randomized controlled trial conducted in a rural, medically underserved Appalachian region to reduce consumption of sweetened beverages was found successful [19], showing that imparting health-related knowledge to school students is an effective method to reach the masses.

Obesity bias in society creates an unhealthy and stressful environment for persons with this condition. It results in fewer work opportunities and may even compromise the level of healthcare received by these individuals [20]. Elimination or reduction of obesity bias is required in all social settings. The modules for obesity bias, conducted before and after the OASP, showed high explicit bias in the pre-test (Table 5). The high scores obtained on the AFAS in the pre-test showed higher anti-fat attitudes. The AFAS showed a highly significant decrease in explicit bias after the OASP (p<0.0001). Higher obesity bias was seen in male students on the AFAS, both before and after the OASP (p<0.0001). BAOP Scale scores were low in the pre-test, indicating a stronger belief that persons who are overweight or obese are responsible for their condition. Pre-test BAOP scores showed no significant difference between female (mean 19.76) and male (mean 19.71) students (p=0.993). Post-test BAOP scores were significantly higher in male (mean 25.92) than in female (mean 25.0) students. The difference between pre- and post-test scores was highly significant in both female and male students, showing the effectiveness of the OASP.

Anti-obesity bias may be present at an early age [10], results in weight-related discrimination, and lowers the self-esteem of the affected person [11]. In our previous work on medical students, our obesity sensitization program lacked dramatization [21]. In this study, we included videos related to obesity discrimination in schoolchildren, in families, and in society. This had a great impact on the audience. Students, faculty, and staff of the schools expressed realization of the harmful effects of weight-related teasing and bullying and promised that they would attempt to stop it. This might help reduce discrimination against persons with obesity in society.

Limitations of the study

This pilot study was conducted on 389 students. A larger sample size would have better emphasized the importance of conducting such health-related programs for school students.

## Conclusions

This study shows the lack of awareness about the causes, consequences, and stigma of obesity in school students. The student participants in this study were informed of the causes of obesity and diseases associated with this condition. Weight-related teasing and bullying are common in children. Students had explicit obesity bias, as apparent from the AFAS and BAOP pre-tests. The sensitization lecture reduced the explicit bias in these students.

The OASP increased the students’ knowledge regarding these issues. This method of reaching out to the students of a community can help spread information rapidly in the community. It also affects the persons directly associated with the medical condition (in this case, obesity and related diseases). We hope that the student participants in this study will be more conscious about their healthy weight, adopt healthy lifestyles to avoid obesity, and develop a more compassionate attitude toward persons with this condition.
